# High throughput measure of diversity in cytoplasmic and nuclear traits for unravelling geographic distribution of rosemary

**DOI:** 10.1002/ece3.4998

**Published:** 2019-03-18

**Authors:** Angelina Nunziata, Laura De Benedetti, Ilaria Marchioni, Claudio Cervelli

**Affiliations:** ^1^ Research Centre for Olive, Citrus and Tree Fruit (OFA) C.R.E.A. Council for Agricultural Research and Economics Caserta Italy; ^2^ Research Centre for Vegetable and Ornamental Crops (OF) C.R.E.A. Council for Agricultural Research and Economics Sanremo Italy; ^3^ Department of Agriculture, Food and Environment (DAFE) Università di Pisa Pisa Italy

**Keywords:** biodiversity, ex situ preservation, genotype confidence percentage, high‐resolution melting, microsatellite, *Rosmarinus officinalis*

## Abstract

In the present work, variability of both cytoplasmic and nuclear microsatellite traits was investigated with the aim of characterizing a set of rosemary germplasm resources (*Salvia rosmarinus*). Most of the materials were collected in Italy and France. High‐resolution melting curves were compared each other computing their Euclidean distances and estimating the differences within their principal component as a measure of genetic diversity. Mantel correlation results combined to linear discriminant analysis allowed examined populations to be divided in four principal groups corresponding to four geographic areas, with few interesting and discussed exceptions. As rosemary propagates by seeds coming from insect mediated pollination, steady wild populations can be expected to be in panmictic equilibrium. Gained results confirmed and extended precedent characterization of rosemary genotypes and are compatible with the distribution of other Mediterranean species, as well as with the presence of a glacial *refugium* in the north‐east area of Sardinia previously described. As the officinal use of this aromatic shrub is spreading, characterization and conservation of wild Mediterranean germplasm is gaining strategic importance. A core collection of 100 genotypes was pointed out as suitable for a cheaper biodiversity ex situ preservation as well as for subsequent metabolic and linkage disequilibrium analyses.

## 1 INTRODUCTION

The common name rosemary is referred to three species of aromatic shrubs native of the Mediterranean Basin that used to be listed in the genus *Rosmarinus* L. (family: *Lamiaceae*,) as *R. officinalis* L., *R. eriocalyx* Jord. & Fourr. and *R. tomentosus* Hub.‐Mor. & Maire. More recently (Drew et al., 2017) the three species were embedded in the genus *Salvia* with the name of *Salvia rosmarinus* Schleid., *Salvia jordanii* J.B.Walker, and *Salvia granatensis* B.T.Drew respectively. *S. rosmarinus* (2n = 24) has a wide natural distribution area, throughout the Mediterranean, and is cultivated all over the world mainly as food flower and ornamental. Otherwise, *S. jordanii* is an Iberio‐Maghrebian endemic species, while *S. granatensis* is an endemic species restricted to the southern part of the Iberian Peninsula (Morales, 2010; Segarra‐Moragues, Carrión Marco, Castellanos, Molina, & García‐Fayos, 2016).

Despite its apparently standard aspect, *S. rosmarinus *is characterized by a variety of growth habits, leaf morphology, flower colors, and scents. It is a pioneer species able to grow on different types of soil and tolerates high temperature and drought as well as poor, dry, sandy, and rocky soil types.

*Salvia rosmarinus* has been intentionally spread beyond its native Mediterranean range to all parts of the world for cultivation as a medicinal, culinary, and ornamental plant. At now, the species is cultivated around the world in both urban and rural gardens and agricultural settings. Cultivation escape and consequent accidental introduction has been reported as well (Randall, 2012). In fact, the species can spread through garden waste or plant parts sold for medicinal and culinary use. It can also be spread by vehicles used in agricultural settings where the species is commercially grown.

In nature, rosemary propagates almost exclusively by seed, that has a short‐distance dispersal because it usually falls and germinates nearby the mother‐plant, sometimes briefly rolling downhill when the plants grow on the cliffs or being transported by ants (myrmecochory; Bouman & Meeuse, 1992). Anthropic propagation is mainly vegetative (by cutting). Rosemary is a monoecy plant with hermaphrodite flowers, on which insect mediated pollination is common both resulting in allogamy and self‐fertilization. Even if rosemary plants are self‐compatible, they often display necessity of cross‐pollination between flowers of the same plant (geitonogamy) due to the strong protandry. Besides, self‐fertilization often leads to inbreeding depression (Hidalgo‐Fernandez & Ubera‐Jimenez, 2001). For these reasons, steady rosemary wild populations can be considered in panmictic equilibrium.

The Romans, Greeks and Egyptians knew beneficial effects of *S. rosmarinus *since ancient times. Ulbricht et al. (2010) have collected and analyzed, in an evidence‐based systematic review of rosemary, a big quantity of scientific literature pertaining to its efficacy in humans, dosing/toxicology, precaution/contraindication (in case of allergy/hypersensitivity to rosemary, pregnancy and lactation and others), adverse effects, interactions and mechanisms of action. This review includes also historical information, expert opinion, historic/folkloric precedents and interesting concluding evidences. To date, although some studies have been done to deeply investigate its medicinal properties (Selmi, Rtibi, Grami, Sebai, & Marzouki, 2017), further research is still required for using rosemary (or its specific constituents) in human therapies.

In the recent period, officinal and cosmetic destinations are becoming economically predominant for this species and the standardization in rosemary growth is an arising issue of the market. Standardization is required in growth protocols and, above all, in essential oil composition and yield. Despite these emerging market instances, traditional rosemary cultivation is a low input agronomic practice in which cultivated varieties are not thoroughly characterized and germplasm resources lack of wide phenomic, biochemical and genomic characterization as well as of database organization. It is known that agronomic practices widely influence essential oil composition, but genotype determines the major part of essential oil composition variability (Li, Cervelli, Ruffoni, Shachter, & Dudai, 2016) as well as genotype‐environment interaction response. Undoubtedly, the characterization of wild rosemary populations could be essential for the identification of one or more genotypes, that may be reflected in one or more chemotypes, able to meet the needs of the market.

Thanks to Mateu‐Andrés et al. (2013), some knowledge about the natural geographic distribution of rosemary genetic variability in western Mediterranean basin is available. The cited work has involved 47 wild populations of *S. rosmarinus* from 10 countries located in this area. Among these, five populations were collected in Italy and five in France. Seven cytoplasmic microsatellites (cpSSR) amplified yielded a total of 17 different alleles; their combination produced 10 different haplotypes. The relationships between haplotypes, their frequencies and geographic distribution were determined, as well as the distribution of haplotypes within populations. The results support the hypothesis that the diversification center of *S. rosmarinus* is in the western part of the Mediterranean basin. The effect of glaciations on Mediterranean plant species, rosemary in particular, and the role of specific areas as refuges for genetic diversity are discussed.

In last 20 years, high‐resolution melting (HRM) analysis was developed for detection of genetic variants in nucleic acid (dsDNA) sequences; HRM a post‐polymerase chain reaction method based on thermal denaturation (melting) (Simko, 2016). The prerequisite for HRM analysis is the use of high‐resolution instruments and new generation saturating dyes (Druml & Cichna‐Markl, 2014; Erali, Voelkerding, & Wittwer, 2008). These latter allow to detect the presence of small insertions or deletions as well as single‐based variants and hence discriminate fragments depending on their sequences. HRM analysis can be performed in plant for many purposes, such as genetic fingerprinting (detection of SNP, SSR and InDels), mapping gene and development of trait‐linked markers, testing food products and seeds (Barcode‐HRM) and genotyping of polyploid species (genome‐wide SNP discovery and detection of allelic dosage). Other emerging application of HRM analysis involve reverse genetic, analysis of cDNA and epigenetic studies (Methylation‐sensitive HRM) (reviewed in Simko, 2016). An example that includes *S. rosmarinus* is the study of Xanthopoulou et al. (2016), who showed a method that involves the use of a nuclear ITS2 gene region for enabling the simultaneous and reliable identification of nine herbal species by multiplex HRM analysis. Within the Lamiaceae family, HRM analysis succeeded in giving an overview of the genetic structure of 19 genebank accessions of *Salvia officinalis *L. (Mader, Lohwasser, Börner, & Novak, 2010): noteworthy is the accordance between some molecular and phytochemical results in the same sample set (Lamien‐Meda et al., 2010; Mader et al., 2010). A few years later, Kalivas et al. (2014) performed the rapid identification and discrimination of seven *Sideritis* species growing in Greece, thanks to the real‐time amplification of ITS2 barcode region coupled with HRM analysis.

In all the cited works, HRM was used to characterize haploid cytoplasmic loci so that HRM profile could always easily be reported to a known allele. In the present work, we characterized genetic variability between and within wild rosemary populations using HRM for both cytoplasmic and nuclear microsatellite markers. A rapid data analysis workflow was used mainly based on measuring Euclidean distance of melting profiles. This method was previously tested as suitable for scoring diversity in heterozygous rosemary (Nunziata, Cervelli, & Benedetti, 2018) and allowed us to monitor a good quantity of loci and genotypes so that statistic elaboration of the data could be robust and reliable. Nonetheless, the method did not imply genotyping so that population genetic analysis was partially limited. These aspect and others concerning strengths and weaknesses of analyzing SSR via HRM have been previously pointed out and discussed (Nunziata et al., 2018).

Aim of the present work was to have a deeper insight on genetic variability of rosemary wild population among Mediterranean basin. For this purpose, Southern Italy genotypes never studied before were included and genomic microsatellite traits were used in combination with cytoplasmic traits previously used. A germplasm collection is conserved ex situ, and available for scientific community at CREA‐OF (Sanremo IM, Italy). This collection is not aimed at preserving the species, that is not in danger of extinction, but in preserving and studying genetic biodiversity within the species itself. Concerning the management of this collection, we needed to establish priority for genotypes to be conserved as well as to define subsets of genotypes on which deeper and wider metabolic analysis should be done.

## 2 MATERIAL AND METHODS

### 2.1 Plant material

Sampling was carried out across the Mediterranean basin to obtain a representative pool of rosemary genotypes. The collection started in 2013 and continued through the years till 2016 and had the main objective of biodiversity preservation and characterization. The 364 individuals tested here have been picked up in 39 natural areas mainly distributed in Tyrrhenian coasts and isles of France and Italy and in Ionian and southern Adriatic coasts of Italy. Eight to ten genotypes have been collected from each area as reported in Supporting Information Table S1. Per each population, genotypes were selected at random, at a minimum distance of 30 m from each other to avoid risks of sampling plants sharing the same parent, taking into account the short distance of seed dispersal. The plants were propagated via stem cuttings and grown in 20 l pots. Four to ten leaves were collected before or after propagation as convenient and lyophilized for subsequent analyses. All the genotypes are conserved ex situ, identified by the codes in Supporting Information Table S1 and available for scientific community at the CREA‐ research center for vegetable and ornamental crop in Sanremo (IM, Italy). One genotype per each considered population is conserved in the Herbarium Mortolense at Giardini Hambury (Ventimiglia, IM—Italy—voucher numbers in Supporting Information Table S1).

### 2.2 High‐resolution melting (HRM) analysis

In order to isolate total DNA from leaf tissues, DNeasy Plant mini Kit (Qiagen—Germany) was used according to manufacturer's instructions. Twenty milligram of lyophilized leaves were disrupted by TissueLyser (Qiagen—Germany) and treated as recommended. Total DNA was eluted in two steps using 70 and 50 µl of elution buffer and collecting both eluates in the same tube. DNA quality and yield were checked out by UV‐vis spectrometer, evaluating absorbance spectra in the wavelength range between 220 and 350 nm. DNA integrity was evaluated on random and bulked samples by agarose gel electrophoresis.

Primer pairs for 16 microsatellite loci were collected from literature (Supporting Information Table S2). Among these primer pairs, four were built on rosemary plastid sequences (ccmp series; Mateu‐Andrés et al., 2013), eight were specifically built on rosemary genomic sequences (Roff series; Segarra‐Moragues & Gleiser, 2009) and four were built on *Salvia officinalis* L. (SoUZ series; Molecular Ecology Resources Primer Development Consortium, 2010). The last four already showed amplification in rosemary (Radosavljević, Jakse, Javornik, Satovic, & Liber, 2011).

For HRM analysis, reactions were built up by Corbett CAS 1,200 robotic instrument in a total volume of 10 µl, using an amount of 5 ng of template DNA per reaction and a final concentration of 50 nM of each primer. SsoFast™ EvaGreen® Supermix (Biorad—USA) was used according to manufacturer's instructions. A touchdown amplification protocol was used decreasing annealing temperature by 0.5 degrees per cycle starting from a temperature 4.5 degree above recommended annealing temperature (*T*_a_) and for 10 cycles. Melting was conducted after denaturing at 90°C and renaturing PCR products at 50°C for 2 min. Acquisition was made during cycling amplification, acquiring luminescence each cycle, and in the denaturation phase, acquiring luminescence each 0.1°C. The size of amplified fragments was verified loading some of the products (random and bulked) on agarose gel.

### 2.3 Data analysis

Data collection and normalization was performed using Rotor Gene 6000 proprietary software (version 1.7.87); the fluorescence (*F*) over temperature (*T*) curve (Wittwer, Reed, Gundry, Vandersteen, & Pryor, 2003) was mainly used. First, normalization was performed: for each marker, normalization ranges were chosen in the regions of pre‐ and postmelting, these regions were chosen as small as possible (1°C, corresponding to 10 data points) and as near as possible but not overlapping the region of melting.

After exporting normalized data in R software (R Core Team, 2016), a similarity matrix for each microsatellite was built up in which each genotype was compared to all the others (values for invalid melting profiles were substituted by the code “na” and excluded from subsequent elaborations). Euclidean dissimilarity was computed as follows:

$$d_{\mathrm{rt}}=\sqrt[{2}]{\sum_{i=a}^{z}{\left(f_{\mathrm{ri}}-f_{\mathrm{ti}}\right)}^{2}}$$

where *f_ri_* and *f_ti_* are the normalized fluorescence values detected at temperature *i* for the *r* and *t* compared samples; *a* is the temperature at the starting point of pre‐melting normalization and *z* is the temperature at the ending point of the post‐melting normalization.

As genotype confidence percentages (GCPs) are similarity values (S) usually computed using the formula: $S_{\mathrm{rt}}=1.05^{\left[-0.02\times d_{\mathrm{rt}}^{2}\right]}$, such transformation of Euclidean dissimilarity was computed (Nunziata et al., 2018) and the corresponding dissimilarity ($D_{\mathrm{rt}}=1-S_{\mathrm{rt}}$) was used for subsequent Mantel correlogram (using vegan package in R) and principal coordinate analysis (PCoA) using DARwin software (Perrier & Jacquemoud‐Collet, 2006).

Also, raw normalized data were exported in DARwin, where an average dissimilarity matrix was built up by Euclidean function (bootstrap value was set to 1,000; Perrier, Flori, & Bonnot, 2003). In these analyses, genotypes having more than 40% missing values were discarded. This last dissimilarity matrix was used for building a bootstrapped Neighbor Joining dendrogram. A core collection of 100 genotypes was also built using Max‐length subtree method (Perrier et al., 2003) by DARwin software.

All these analysis (average transformed dissimilarity matrix, Mantel correlogram, PCoA, bootstrapped untransformed dissimilarity matrix and dendrogram building) were also performed considering exclusively the data concerning ccpm series microsatellite.

To better describe and analyse diversity of melting profiles of each molecular marker in each genotype, PAST (Hammer, Harper, & Ryan, 2001) was used to make principal component analysis (PCA) on the fluorescence data per temperature from each satellite. 16 PC1 scores (deriving from the analysis of all the microsatellite) and 12 PC2 scores (deriving only from the nuclear microsatellite) were transformed adding the constant value *k *= 120 and used as independent variables for subsequent linear discriminant analyses. LDA was performed at first grouping genotypes by populations, and then repeated twice grouping genotypes according to first results as better specified hereafter. All the three LDA were also performed only using the four PC1 scores coming exclusively by cytoplasmic markers in order to have indication about diversity in pollen and seed dispersion.

## 3 RESULTS

Results were observed in Rotor Gene Software both by “melting analysis” modality and by “HRM analysis” modality. A total of 5,824 melting curves with their respective amplification profiles were observed, coming from the amplification of 364 different samples by means of 16 primers pairs. Among these 5,824 melting curves, 5,670 (97.4%) were considered a result of successful and specific amplification as they were amplified with the same CT (number of amplification cycle in which the amplification signal started arising). A glance to the melting curves confirmed that all those gained from each primer pair were comparable (similar *T*_m_ and shape; raw data in https://doi.org/10.17632/bmxm4vvxdp.1). 154 (2.6%) curves that came from inefficient or not specific fragment amplification were therefore scored as not available data. The observation of the reaction ring after run and during first screening of results revealed that inefficient amplification was mainly due to insufficient sample loading and/or inefficient adhesion of film cover. The presence of secondary peaks the major part of HRM profiles deriving from nuclear traits confirmed a high level of allogamy in analyzed genotypes.

**Figure 1:**
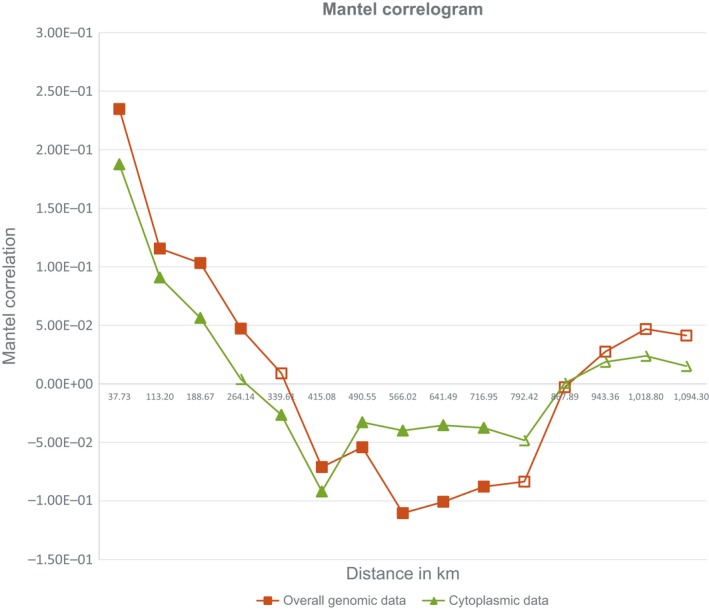
Mantel correlogram. Bold points indicate values with statistical significance major than 99%

The 66,066 computed average transformed Euclidean distances (*D_rt_*) ranged from 0.08 to 0.95, with a global average value of 0.57. From these distances 38 average intra‐population and 703 average inter‐population genetic distances were computed. The sample PORTO was excluded from this analysis. The intra‐population genetic distances, corresponding to a geographic distance of 0 km, ranged from 0.22 (CIR population) to 0.56 (GON population) and showed an average value of 0.34 with a standard deviation of 0.10. A negative (slope = −9 × 10^−5^) but weak (*R* = 0.1488) correlation was found between these values and the respective distance from the diversification center of the species localized in the southern east of the Hiberian peninsula by Mateu‐Andrés et al. (2013). The 703 average inter‐population genetic distances ranged from 0.28 to 0.82, with an average value of 0.58 and a standard deviation of 0.11. The corresponding approximate geographic distances ranged from 17 km to 2,173 km with an average value of 541 km and a standard deviation of 361 km. Mantel correlogram evidences an exponential‐like decease of correlation index. The geographic distance at which the Mantel correlation is zero is about 340 km if all SSR results are considered, 270 km if only cytoplasmic distances are taken in account (Figure 1). In all the 741 examined populations pairs the cytoplasmic contribution to average genetic diversity ranged from 3% to 33%, with an average value of 18% and a standard deviation of 4%. No correlation was found between geographic distance and cytoplasmic contribution to genetic distance.

**Figure 2:**
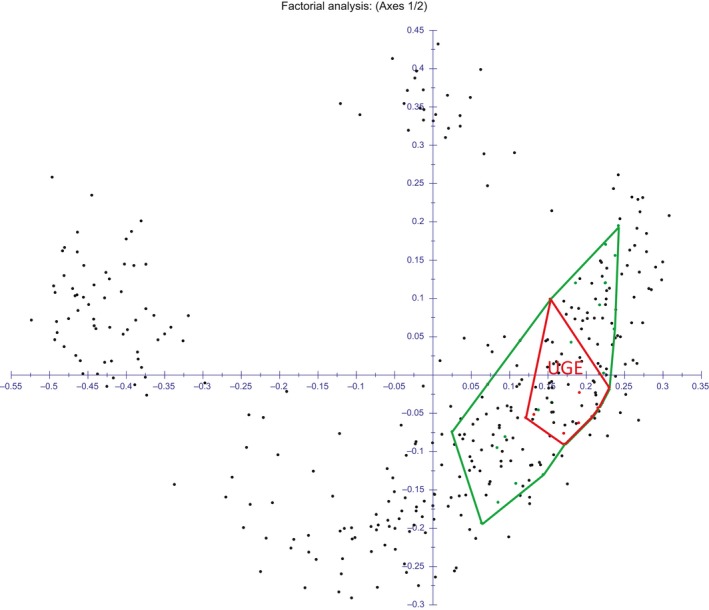
Principal Coordinates Analysis made using average transformed Euclidean distances among melting curves as a measure of genetic distances. The first two coordinates are indicated on the two axes. Genotypes of the population UGE have been included in the red polygonal. The polygonal including all genotypes collected within 130 km has been marked in green

PCoA based on transformed Euclidean distances (*D_rt_*) (Supporting Information Figure S1), revealed as genotypes belonging to the same population could almost all be localized in a region of the scattergram and genotypes coming from the same geographic region tended to cluster in adjacent regions of the scattergram. As an example, in Figure 2, the minimal convex polygonal including all genotypes belonging to the population UGE (collected in Marina di Ugento, Puglia) has been marked in red and the polygonal including all genotypes collected within 130 km from Marina di Ugento (populations UGE, CESI and CHIA) has been marked in green. Noteworthy, all the southern Italy genotypes could be found in the right lower part of the graph. The low representation of the first two axis (axis 1 = 16.56% and axis 2 = 7.47%) and the dispersion of the genotypes in the first axes (Supporting Information Figures S1 and S2) reveal a low level of diversity in population structure.

Neighbor Joining tree built using Euclidean untransformed average distances (Supporting Information Figure S2) evidenced as a main branch could be identified comprising all the genotypes collected in southern Italy, another, less numerous, comprising all genotypes from France and Liguria (green), while two branches comprised genotypes coming from Sardinia, Corse and Tuscany (orange). More dispersed, genotypes from Spain and Elba Isle formed minor branches of the tree. The bootstrap value evidences that the two identified main Sardinian/Corse/Tuscany branches have a solid structure. Among the other branches, instead, the bootstrap values were high only for groups formed by genotypes of the same population or by genotypes coming from populations very close each other (Supporting Information Figure S2).

The PCA made on the fluorescence data from each satellite allowed to evidence a first principal component, which is explained by the shifting of the melting temperature of the fragment and, only for the nuclear marker, a second principal component, which substantially describes the degree of heterozygosity (presence of secondary flex points). So, 16 PC1 scores (deriving from the analysis of all the microsatellite) and 12 PC2 scores (deriving only from the nuclear microsatellite) were transformed and used as independent variables for subsequent linear discriminant analyses.

These variables explained, on average, 95.35% of observed variability.

Linear Discriminant Analysis of genetic characterization of 364 rosemary genotypes grouped by populations evidenced the existence of three principal nest (Supporting Information Figure S3).

The confusion matrix (Supporting Information Table S3) evidenced as 89.56% of individuals were correctly assigned to each population basing on the value of the 28 considered variables (16 PC1 + 12 PC2). Besides, 8 populations appeared well discriminated from all the other samples as no false positive nor false negative were scored in the confusion matrix. These were CESI, CHIA, and MATI, collected in Puglia, MAR and SPIE, collected in Sardinia, MAC from Latium, POS from Campania and TOR from Sicily.

Looking at the geographic origins of the population in each of the three nests, it is evident that the populations in the nest on the left part of the scattergram are population collected in Sardinia, Corse and Tuscany. Populations in the right superior part of the scattergram were almost all collected in Liguria and Continental France, populations in the right lower part were collected in Southern Italy and Sicily.

Few exceptions can be observed:

1. FET population, coming from Elba Island (Tuscany), and TOR population coming from Sicily, apparently clustered within Liguria/France group.
2. MAR and SPIE populations, coming from south west of Sardinia, apparently clustered within Southern Italy Group.

As told, MAR, SPIE and TOR population are 100% genetically distinguishable from all the other populations (Supporting Information Table S3) while FET is 95% due to one false positive (coming from Liguria). Interestingly, DON population, collected in Andalucía (Spain), was observed together with the PORTO genotype, collected in Portugal, in the central part of the scattergram, apart from all the others.

**Figure 3:**
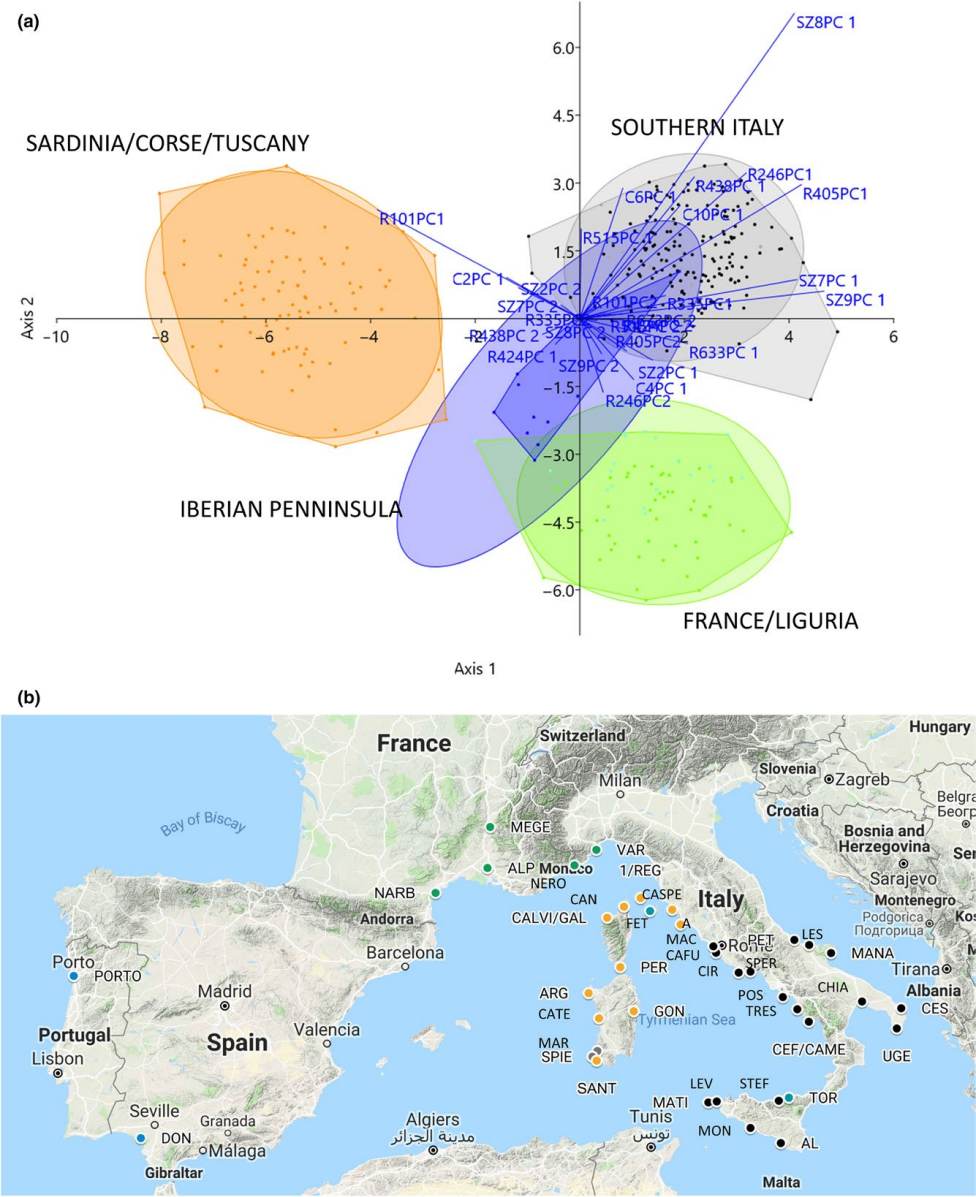
Panel a shows third linear discriminant analysis (LDA) of genotypes divided in four groups as emerged from first LDA. Panel b shows on the map the points in which population grouped in the four groups have been collected. The colors of the points correspond to the four colors of the groups in LDA

A second LDA was conducted grouping individuals according to their actual geographic origin divided in four regions: Southern Italy, Sardinia/Corse/Tuscany, Liguria/France and Iberian Peninsula. In this second LDA, the percentage of correctly classified genotypes arises to 94.2% as expected reducing the number of groups. In order to verify if the forced attribution to genetic groups of FET, TOR, MAR and SPIE population was incorrect, a third LDA was also conducted grouping genotypes as they appeared grouped in the three nests and the central part in the first LDA (Figure 3a). In this analysis the percentage of correctly classified genotypes raised to 98.9%. As shown in the confusion matrix in Table 1, the three nest groups showed three false negative (one included in the wrong nest, and two included in the Iberian Peninsula central group) and one false positive (coming from the central group).

**Table 1:** Confusion matrix coming from third linear discriminant analysis

	Predicted as Southern Italy	Predicted as Sardinia/Corse/Tuscany	Predicted as France/Liguria	Predicted as Iberian Peninsula	Total genotypes in the group
Southern Italy	195	0	1	1	197
Sardinia/Corse/Tuscany	0	87	0	1	88
France/Liguria	0	0	68	0	68
Iberian Peninsula	1	0	0	10	11
Total predicted genotypes	196	87	69	12	364

Figure 3b shows the map including the 39 points in which genotypes have been collected. In this map, populations included in the four groups show different colors according to the nest in which population can be localized in LDA. As evident, clustering is consistent with geographic origin of populations.

All the analysis conducted only using ccpm markers confirmed clustering obtained by means of all the markers, but with a predictable greater instability of branches in the dendrograms and of group prediction in LDA (data not shown).

**Figure 4:**
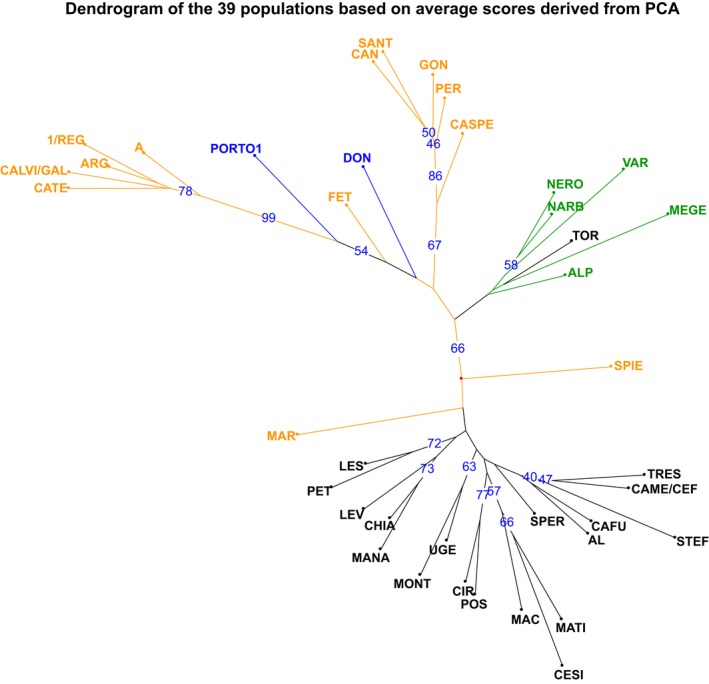
Neighbor Joining tree of 39 populations. Principal components of melting curve of each markers have been transformed, averaged in each population and used as variable

A dendrogram was also built using average PC scores of the 39 population (Figure 4), to focus on relationships between considered populations. This tree confirmed the overall diversity between southern Italy group, Liguria‐France group and Sardinia/Corse/Tuscany. Some association were also evidenced between geographically close populations in southern Italy (LES‐PET; CHIA‐MANA and CIR‐POS). In Sardinia Corse group, dichotomy was confirmed and Thirrenian populations seemed to form a separate cluster from the other populations. Comparing these two last clusters to the other gained in the dendrogram built on 364 samples data, it must be evidenced that not all the genotypes of each population are in the same main cluster, confirming that Sardinia and Corse populations are particularly rich of biodiversity.

**Figure 5:**
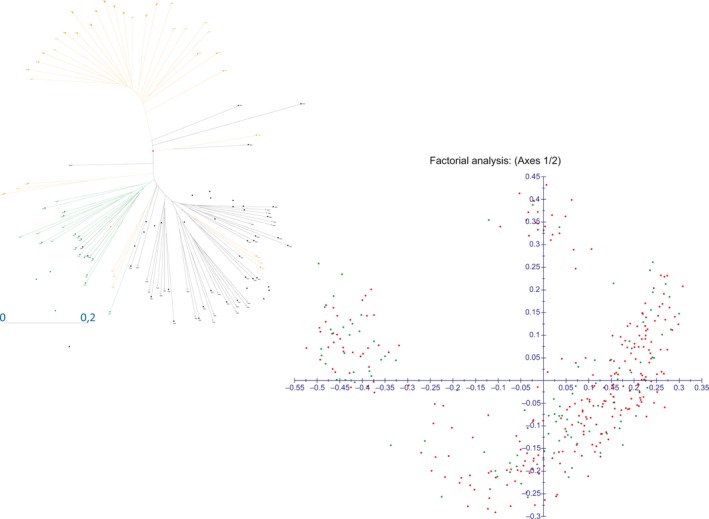
Description of genetic diversity of the 100 genotypes Core collection pointed out. In LDA Kept genotypes are represented in green and removed ones are in red

Results for a core collection including 100 individuals, suitable for subsequent Linkage disequilibrium studies, are shown in Figure 5. Genotypes included in the core collection are distributed in all populations, including from 0 to 6 genotypes per population, with most of them being represented by two or three genotypes (Supporting Information Table S4). Populations CIR, FET and SPER are not represented in the core collection but kept genotypes are uniformly distributed in the four cluster as evidenced by the scattergram in Figure 5.

**Figure 6:**
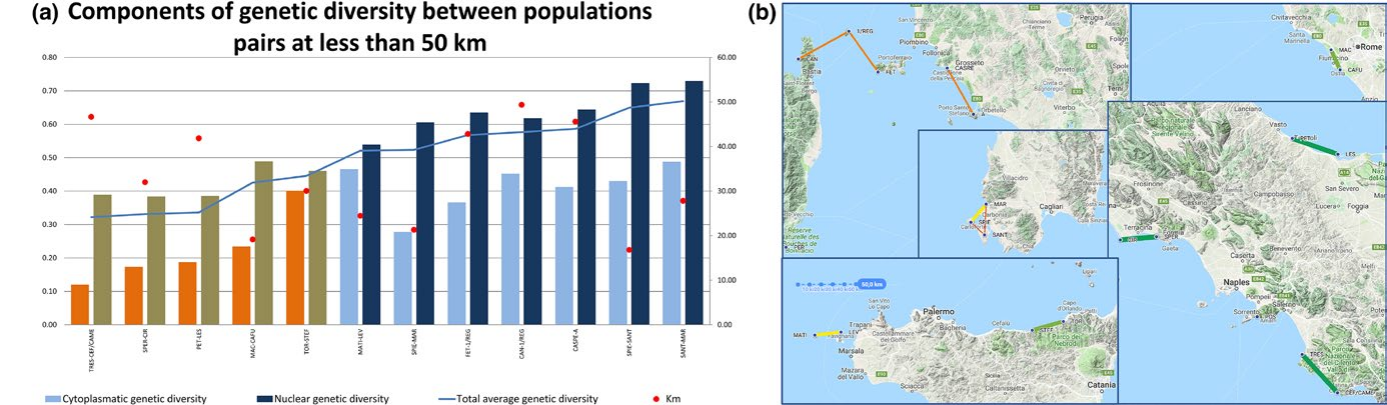
Genetic distances between population distant less than 50 km from each other. In panel a nuclear, genetic and cytoplasmic distances are represented. Population interspaced by land are in orange/brown, population interspaced by sea channels are represented in blue/light blue. In panel b the populations are shown on the map

Deeper insights came from the comparison of genetic distances measured by cytoplasmic and nuclear markers in 12 pairs of populations very close each other (less than 50 km; Figure 6a,b).

In these 12 pairs, a higher average genetic distance was found in the seven populations pairs divided by a sea channel. Besides, in four of the five populations pairs divided by land, cytoplasmic genetic distance was particularly low, above all in the population pair TRES‐CEF/CAME (only 9% of the total detected diversity), as expected between populations originated from geographic isolation of groups from the same original population. On the other hand, the populations pairs TOR‐STEF (separated by land) and MATI‐LEV (separated by sea) were characterized by a relatively high cytoplasmic genetic distance (22% of the total detected diversity), as expected when seeds or vegetative material imported from a different geographic region give rise to a new population.

## 4 DISCUSSION

Rosemary pollination is mediated by insects of several orders (*Hymenoptera apoidea*, *Lepidoptera* in general and *Sphingidae* in particular) and even hummingbirds have been observed while visiting rosemary flowers outside from the natural Mediterranean distribution area.

Herrera (2005) reported that a great potential to local differentiation exists in *Rosmarinus *due to corolla size variability. Our data show that there is relatively high Mantel correlation in the first distance classes, which tend to decrease and stabilize after a given distance class, indicating that there are patches of genetic variation or similarity. The geographic distance at which the Mantel correlation is zero (340 km) indicates the size of the patch (Diniz‐Filho et al., 2013). The presence of such patches of genetic isolation could be due to patches of different insect distribution.

In the comparison of genetic distances measured in the 12 pairs of nearest populations (Figure 6a,b), a higher average genetic distance was found in the seven populations pairs divided by a sea channel confirming that the presence of sea largely reduces the pollen flow between populations as the major part of pollinators are not able to cross sea channels. In general, populations separated by sea show a major degree of nuclear traits differentiation as a confirm of the pollen flow interruption.

In the above‐mentioned work by Mateu‐Andrés et al. (2013), 10 of the 47 examined populations were collected in Italy and France. Among these, H2 and H4 were the most detected haplotype. According to the authors, all of the 10 population belonged to the same cluster of genetical diversity, with some populations collected in Spain and Portugal forming another cluster. Our results are perfectly compatible with those of Mateu‐Andrés et al. (2013), who evidenced the prevalence of H4 haplotype (and its derivate H5 and H6) in continental France, the prevalence of H2 haplotype in southern Italy and south Sardinia, and a mixture of H4, H2 and H1 in the two Corse populations. Nonetheless in the present work, due to a major number of marker analyzed, a more sensitive detection technique and a more detailed sampling in Italy and France, the presence of three genetic clusters in this region was demonstrated, as well as a major richness in rosemary biodiversity in Sardinia/Corse region compared to the rest of France and Italy.

The presence of two distinct sub‐clades among Sardinian population is compatible with previous findings in other Mediterranean species by Melito et al. (2013) who reported a similar distribution of genetic populations in *Helichrysum italicum* Roth. Like rosemary, *Helichrysum* is an allogamous plant whose pollen transport is mediated by insects. In another work, Medail and Diadema (2009) reported the presence of a glacial *refugium* for plants in the north‐east area of Sardinia (*refugium* n°19). The presence of this area of biodiversity storage can explain the diversity we found among Sardinian populations in the present study.

The nature of the data gained in this study does not allow to establish the age in which direct connections have occurred between populations SPIE and MAR (in S. Pietro island and Fontanamare, south west of Sardinia) and the other southern Italy rosemary populations. Further analysis on coding sequences could help understanding, as an example, if the boundaries of the Carthaginian Empire in 400 a.C. or more recent events in the peculiar history of S. Pietro island between Genoa and Tunis have determined this exception in homogeneous geographic genetic distribution.

Regarding the clustering of FET population, coming from Elba Island (Tuscany), and TOR population coming from Sicily, in the France/Liguria group, many hypotheses can be done. The two populations share an uncommonly high similarity at cytoplasmic level (90%) that could be explained only by a movement of seeds or other vegetative parts between the cited places. Rosemary seeds are not known as principal seeds in bird nutrition, but sparrow‐like birds have been observed while eating seeds on rosemary bushes. In particular, house finches (H*aemorhous mexicanus, *P.L.Statius Muller, 1776) have been observed eating rosemary seeds in Georgia (USA) and goldfinches (*Carduelis carduelis *L., 1758) in Texas (USA). Searching for migration routes of sparrow‐like birds in Italy, we found that tree pipits (*Anthus trivialis *L. 1758), yellow wagtails (*Motacilla flava *L., 1758) and European serins (*Serinus serinus *L., 1766) have been observed moving between Liguria and Sicily (Spina & Volponi, 2008, pp 86, 108, 520). Among these, tree pipits and yellow wagtails are prevalently insectivore, but European serins are granivore and could act as rosemary seed carriers. European goldfinches (*Carduelis carduelis *L., 1758) have been observed moving along Thirrenian coast as well, but not specifically between Liguria and Sicily (Spina & Volponi, 2008, p 540). A seed movement from Liguria and Elba to Torrenova (in Sicily), could also explain the high cytoplasmic diversity observed between the genotypes collected in Torrenova and the genotypes collected less than 50 km away in Santo Stefano di Camastra. As sparrow‐like birds move widely along Mediterranean coasts, it remains unclear why this flux of genetic material has been so efficient compared to what happened along other possible routes. Regarding genotypes collected in Torrenova, it must be specified that those were the only ones collected along the path of a stream. This could explain an enhanced efficiency of a hypothetic seed carriage mediated by European serins, but this could also lead to the hypothesis that this particular population could come from an accidental cultivation escape. Genetic analyses on local rosemary cultivated varieties could help discerning this case.

Among the 39 populations here analyzed, 13 had already been characterized for their volatile composition in a previous work (Li et al., 2016): two populations from Sicily (STEF and AL), one from Levanzo isle (LEV), three from Sardinia (MAR and ARG), three from Corse (CAN and CALVI/GAL), one from Caprera island (1/REG), two from Tuscany (A and CASPE) and one from Andalucia (DON). Chemical composition evidenced that the Corse and the Sardinian genotypes grouped together lacking both Sesquitherpene Hydrocarbons. Differently from our findings, the Tuscan and the Sicilian genotypes were grouped both in the Chemiotype A. Nonetheless, the Tuscan genotypes showed a production of Bornyl‐Acetate generally higher than the Sicilian ones. Besides, some discrepancies are expected as differences observed in the present work are presumably due to overall genetic diversity while volatile composition could be a consequence of selective pressures driving in convergent evolutionary directions.

Regarding the management of the rosemary collection in our institute, we evidenced the presence of many important genotypes different from others already described in other works and collected elsewhere. In particular, the genetic characterization evidenced that southern Italy populations are very different from rosemary collected elsewhere and feature this particular collection. Building of core collection can be the first fundamental step for a correct study of statistical association between morpho‐physiological traits and genetic markers. A core collection should be characterized by the maximum possible genome‐wide variability and by lack of structure, that should ensure low statistical association between randomly chosen distant traits (Frankel & Brown, 1984). As reported by Garcia‐Lor, Luro, Ollitrault, and Navarro (2017), ML subtree method is suitable for building core collections from allogamous plant collections and has better performances than other five tested methods when the number of individuals is reduced to the 18%–41% of the initial number of genotypes. Our core collection falls within these percentages, as the number of genotypes was reduced to 27%. Anyway, in order to ensure storage of specific interesting morpho‐physiological traits, other parameters should be considered. In conclusion, the core collection described in Figure 5 is suitable for subsequent metabolic and linkage disequilibrium analyses and can be stored and deeply investigated with less expenses.

## ANTIPLAGIARISM DECLARATION AND CONFLICT OF INTEREST

The authors declare that this piece of written work is the result of their own independent scholarly work, and that in all cases material from the work of others is acknowledged, and quotations and paraphrases are clearly indicated. No material other than that listed has been used. This written work has not previously yet been published. The authors declare that they have no conflict of interest.

## AUTHORS CONTRIBUTION

A.N. conceived and designed the study. C.C. performed population retrieval and sampling across Mediterranean. L.D.B. and I.M. performed DNA extraction and HRM. A.N. performed data analyses. All the authors participated in writing the manuscript.

## Data Availability

The data consisting of 5,824 normalized melting profiles of sixteen SSR fragments of 364 individuals are provided in Nunziata, Angelina; De Benedetti, Laura; Marchioni, Ilaria; Cervelli, Claudio (2019), “High Resolution Melting profiles of 364 genotypes of Salvia rosmarinus in 16 microsatellite loci,” Mendeley Data, v1 http://dx.doi.org/10.17632/bmxm4vvxdp.1.
