# Supplementary figures and images for: High throughput measure of diversity in cytoplasmic and nuclear traits for unravelling geographic distribution of rosemary
Source: Ecol Evol. 2019 Mar 18;9(7):3728–39. doi: 10.1002/ece3.4998 (PMC6468057; doi:10.1002/ece3.4998)

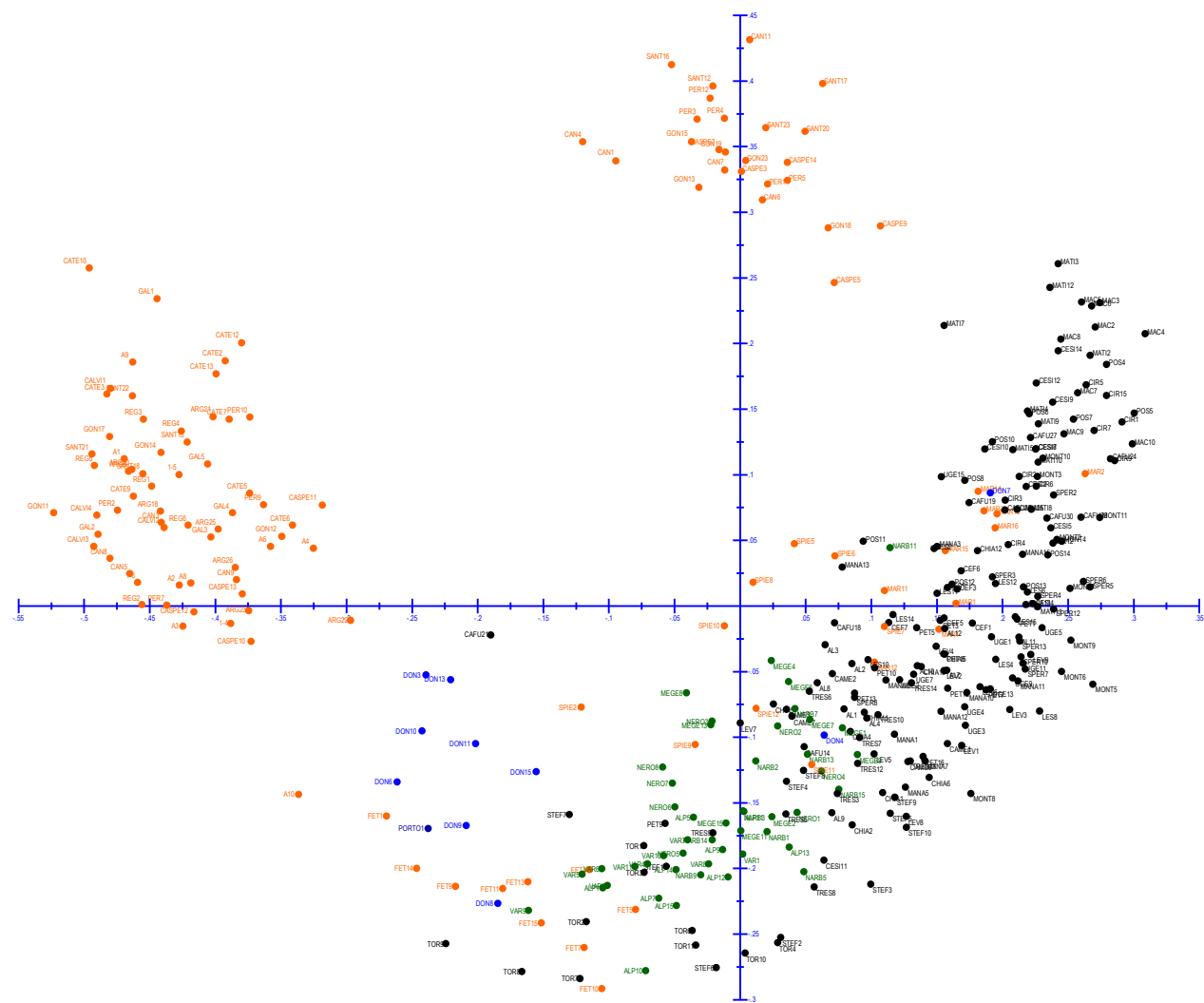

Supplement: Supplementary file 1 [file ECE3-9-3728-s001.pdf]

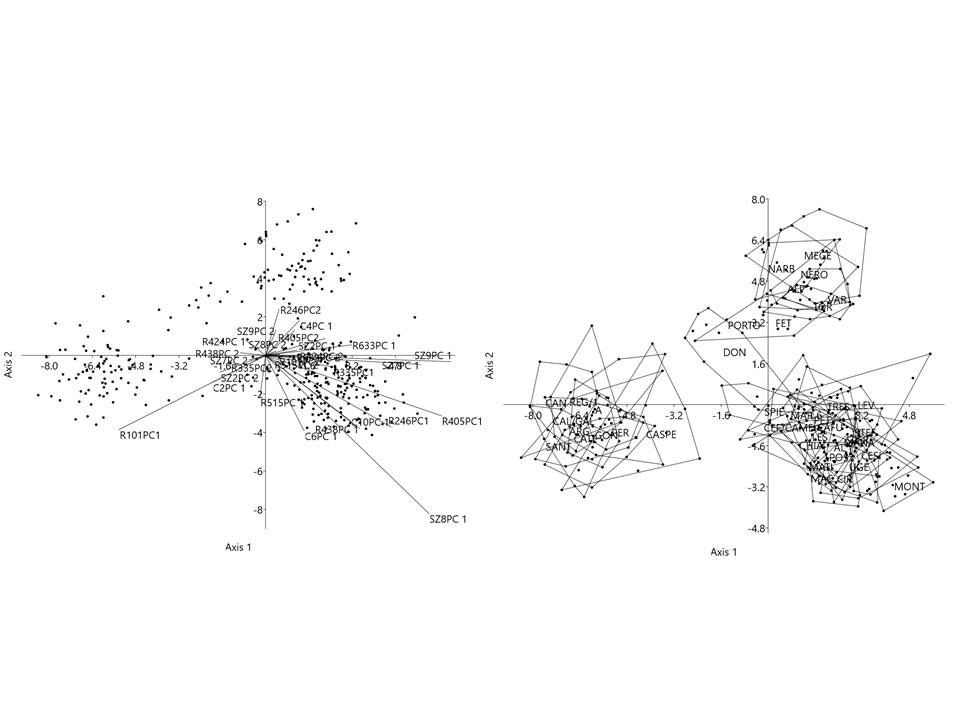

Supplement: Supplementary file 4 [file ECE3-9-3728-s004.jpg]
