# Supplementary material for: High throughput measure of diversity in cytoplasmic and nuclear traits for unravelling geographic distribution of rosemary
Source: Ecol Evol. 2019 Mar 18;9(7):3728–39. doi: 10.1002/ece3.4998 (PMC6468057; doi:10.1002/ece3.4998)
Supplement: Supplementary file 5 [file ECE3-9-3728-s005.docx]

Supplementary table 1: Codes, region and coordinates of rosemary sampled populations.

| Population | Region | Latitude | Longitude | Locality | # genotypes^[[1]](#footnote-1)^ | Herbarium voucher code |
| --- | --- | --- | --- | --- | --- | --- |
| **PORTO** | Portugal | 41.15794 | -8.6291 | Porto | 1 | HMGBH.e/7219.2018.029 |
| **DON** | Spain | 37.04272 | -6.43444 | Matalascanas | 10 | HMGBH.e/7219.2018.013 |
| **NARB** | France | 43.16192 | 3.16805 | Narbonne | 10 | HMGBH.e/7219.2018.025 |
| **ALP** | France | 43.75772 | 4.8512 | Mouries | 10 | HMGBH.e/7219.2018.003 |
| **MEGE** | France | 44.71355 | 4.944 | Frontignan | 10 | HMGBH.e/7219.2018.023 |
| **NERO** | Liguria | 43.81475 | 7.69354 | Ospedaletti | 8 | HMGBH.e/7219.2018.026 |
| **VAR** | Liguria | 44.1822 | 8.40243 | Varigotti | 10 | HMGBH.e/7219.2018.039 |
| **FET** | Tuscany minor isles | 42.73432 | 10.14671 | Pomonte – Elba Isle | 10 | HMGBH.e/7219.2018.014 |
| **1/REG** | Tuscany minor isles | 43.04785 | 9.84104 | Capraia Isle | 9 | HMGBH.e/7219.2018.031 |
| **CASPE** | Tuscany | 42.76389 | 10.87502 | Castiglione della Pescaia | 9 | HMGBH.e/7219.2018.007 |
| **A** | Tuscany | 42.40726 | 11.15041 | Monte Argentario | 8 | HMGBH.e/7219.2018.001 |
| CALVI/GAL | Corsica | 42.56765 | 8.75722 | Galeria | 9 | HMGBH.e/7219.2018.015 |
| CAN | Corsica | 42.83329 | 9.30974 | Punta di Canelle | 9 | HMGBH.e/7219.2018.006 |
| PER | Corsica | 41.366 | 9.18083 | Capo Pertusato | 9 | HMGBH.e/7219.2018.027 |
| ARG | Sardinia | 40.73386 | 8.1483 | Alghero-Argentiera | 8 | HMGBH.e/7219.2018.004 |
| CATE | Sardinia | 40.10737 | 8.49116 | Santa Caterina di Pittinuri | 9 | HMGBH.e/7219.2018.008 |
| MAR | Sardinia | 39.28731 | 8.43555 | Fontanamare | 10 | HMGBH.e/7219.2018.021 |
| GON | Sardinia | 40.28035 | 9.62717 | Cala Gonone | 9 | HMGBH.e/7219.2018.016 |
| SANT | Sardinia | 39.03739 | 8.41842 | Sant'Antioco Isle | 9 | HMGBH.e/7219.2018.032 |
| SPIE | Sardinia minor isles | 39.14071 | 8.276 | San Pietro Isle | 9 | HMGBH.e/7219.2018.034 |
| **CAFU** | Latium | 41.72067 | 12.30871 | Castel Fusano | 10 | HMGBH.e/7219.2018.005 |
| **SPER** | Latium | 41.26367 | 13.42714 | Sperlonga | 10 | HMGBH.e/7219.2018.033 |
| **MAC** | Latium | 41.87774 | 12.21359 | Macchiagrande | 9 | HMGBH.e/7219.2018.019 |
| **CIR** | Latium | 41.23816 | 13.04575 | Monte Circeo | 10 | HMGBH.e/7219.2018.012 |
| **TRES** | Campania | 40.32779 | 14.95735 | Monte Tresino | 10 | HMGBH.e/7219.2018.037 |
| **POS** | Campania | 40.62805 | 14.48498 | Positano | 10 | HMGBH.e/7219.2018.030 |
| **CEF/CAME** | Campania | 40.01947 | 15.33031 | Marina di Camerota | 10 | HMGBH.e/7219.2018.009 |
| **PET** | Molise | 42.03563 | 14.85531 | Marina di Petacciato | 10 | HMGBH.e/7219.2018.028 |
| **CHIA** | Puglia | 40.52152 | 17.06132 | Chiatona | 10 | HMGBH.e/7219.2018.011 |
| **LES** | Puglia | 41.91298 | 15.33415 | Lesina Marina | 10 | HMGBH.e/7219.2018.017 |
| **MANA** | Puglia | 41.71061 | 16.04908 | Mattinata | 10 | HMGBH.e/7219.2018.020 |
| **CESI** | Puglia | 40.3533 | 18.33743 | Strada per le Cesine | 10 | HMGBH.e/7219.2018.010 |
| **UGE** | Puglia | 39.84898 | 18.18962 | Riva di Ugento | 10 | HMGBH.e/7219.2018.038 |
| AL | Sicily | 36.91448 | 14.41431 | Vittoria | 10 | HMGBH.e/7219.2018.002 |
| MONT | Sicily | 37.30787 | 13.42955 | Torre di Monterosso | 10 | HMGBH.e/7219.2018.024 |
| STEF | Sicily | 38.01413 | 14.34795 | Santo Stefano di Camastra | 10 | HMGBH.e/7219.2018.035 |
| TOR | Sicily | 38.08704 | 14.67837 | Torrenova | 10 | HMGBH.e/7219.2018.036 |
| MATI | Sicily minor isles | 37.97397 | 12.05469 | Marettimo Isle | 10 | HMGBH.e/7219.2018.022 |
| LEV | Sicily minor isles | 37.99968 | 12.33207 | Lèvanzo Isle | 9 | HMGBH.e/7219.2018.018 |
|  |  |  |  |  | 364 |  |

1. Only genotypes included in data elaboration are computed [↑](#footnote-ref-1)
