# Supplementary material for: High throughput measure of diversity in cytoplasmic and nuclear traits for unravelling geographic distribution of rosemary
Source: Ecol Evol. 2019 Mar 18;9(7):3728–39. doi: 10.1002/ece3.4998 (PMC6468057; doi:10.1002/ece3.4998)
Supplement: Supplementary file 6 [file ECE3-9-3728-s006.docx]

|  |  |  | Motive | Ta | Fragment size range |
| --- | --- | --- | --- | --- | --- |
| Ccmp2 | ccmp2F | GATCCCGGACGTAATCCTG | (A)_11_ | 50 | 215-218 |
|  | ccmp2R | ATCGTACCGAGGGTTCGAAT |  |  |  |
| Ccmp4 | ccmp4F | AATGCTGAATCGAYGACCTA | (A)_11_ | 50 | 133-134 |
|  | ccmp4R | CCAAAATATTGGGAGGACTCT |  |  |  |
| Ccmp6 | ccmp6tailF | *TGTAAAACGACGGCCAGT*CGATACATATGTAGAAAGCC | (T)_11_ | 50 | 60 -61 |
|  | ccmp6R | CATTACGTGCGACTATCTTC |  |  |  |
| Ccmp10 | ccmp10tailF | *TGTAAAACGACGGCCAGT*TTTTTTTTTAGTGAACGTGTCA | (T)_11_ | 50 | 100-101 |
|  | ccmp10R | TTCGTCGDCGTAGTAAATAG |  |  |  |
| Roff101 | Roff101R | ATTCTTCTCCGAACGAACAGTAG | (GAA)_21_ | 56 | 141-212 |
|  | Roff101V | CATTGTGGCCATGTGAATAGA |  |  |  |
| Roff246 | Roff246F | ATAAACGTAAAGCACCCAACC | (CT)_9_(CA)_7_ | 56 | 107-165 |
|  | Roff246R | AGGTGTGTAGAGAGGGAGAATG |  |  |  |
| Roff335 | Roff335tailF | *TGTAAAACGACGGCCAGT*CTCCGCCAATACCCTTAACCTC | (CT)_9_ | 56 | 99-105 |
|  | Roff335R | CAACGGCAGCGATAGCAGAT |  |  |  |
| Roff405 | Roff405F | TATCACACTCCCACCACCTTCT | (CT)_20_ | 56 | 117-129 |
|  | Roff405R | ATGACTGCCTGCTTCAATCTTC |  |  |  |
| Roff424 | Roff424F | AGATGAAGATGGGTGAACTGAAGA | (GA)_8_ | 56 | 128-150 |
|  | Roff424R | TTGAAGGGTGCATTTGGATAGA |  |  |  |
| Roff438 | Roff438tailF | *TGTAAAACGACGGCCAGT*TGTCGAACAAGAAACTTTAATTCTG | (CT)_12_(CA)_9_ | 56 | 73-109 |
|  | Roff438R | GTCGTCACATCAACTGCCTCT |  |  |  |
| Roff515 | Roff515F | GCTCTCGAGTCACCGTAACCA | (CT)_9_(CA)_4_ | 56 | 144-186 |
|  | Roff515R | CTGGCCACAACGAAATTCAAG |  |  |  |
| Roff633 | Roff633F | TTTGGCTTGCTCTTCATCATTT | (CTT)_12_ | 56 | 137-203 |
|  | Roff633R | CCCCGTAGTAAGATTTATCGTTTG |  |  |  |
| SoUZ002 | SoUZ002tailF | *TGTAAAACGACGGCCAGT*CGTGTCGCTACAAGACAACC | (TG)_11_^^[[1]](#footnote-1)^^ | 55 | 199-222^^[[2]](#footnote-2)^^ |
|  | SoUZ002R | CGCACTCACTCTCTCCCTCT |  |  |  |
| SoUZ007 | SoUZ007F | gcaatcaatgcaagtcagga | (GT)_11_^a^ | 55 | 214-227^b^ |
|  | SoUZ007R | TTGGCTCTAATTATGGGGTCA |  |  |  |
| SoUZ008 | SoUZ008F | TGGAGATTTTGGTGGCATTT | (TC)_15_^a^ | 55 | 190-210^b^ |
|  | SoUZ008R | ACATTTGGCACCATTGAAAA |  |  |  |
| SoUZ009 | SoUZ009F | CTTGTGGTTGCAGAAGGTGA | (TG)_15_^a^ | 55 | 209-250^b^ |
|  | SoUZ009R | CTCCACACACTCCACCATTG |  |  |  |

**Supplementary table 2: Primer pairs as used in this work: in some primer pairs, a tail reported in italic has been added to the sequences previously published. Data regarding motive and expected** **fragment size are reported from literature and refer to fragments without M13 tail**

1. As sequenced in *Salvia officinalis* [↑](#footnote-ref-1)
2. As observed in S*alvia officinalis* [↑](#footnote-ref-2)
